# Biochemical and Taxonomic Characterization of Novel Haloarchaeal Strains and Purification of the Recombinant Halotolerant α-Amylase Discovered in the Isolate

**DOI:** 10.3389/fmicb.2020.02082

**Published:** 2020-09-01

**Authors:** Dipesh Kumar Verma, Gunjan Vasudeva, Chandni Sidhu, Anil K. Pinnaka, Senthil E. Prasad, Krishan Gopal Thakur

**Affiliations:** ^1^G. N. Ramachandran Protein Centre, Structural Biology Laboratory, Council of Scientific and Industrial Research-Institute of Microbial Technology, Chandigarh, India; ^2^MTCC-Microbial Type Culture Collection and Gene Bank, CSIR-Institute of Microbial Technology, Chandigarh, India; ^3^Biochemical Engineering Research and Process Development Centre, Council of Scientific and Industrial Research-Institute of Microbial Technology, Chandigarh, India

**Keywords:** haloarchaea, amylase, pangenome, carotenoids, halotolerant enzyme

## Abstract

Haloarchaea are salt-loving archaea and potential source of industrially relevant halotolerant enzymes. In the present study, three reddish-pink, extremely halophilic archaeal strains, namely wsp1 (wsp-water sample Pondicherry), wsp3, and wsp4, were isolated from the Indian Solar saltern. The phylogenetic analysis based on 16S rRNA gene sequences suggests that both wsp3 and wsp4 strains belong to *Halogeometricum borinquense* while wsp1 is closely related to *Haloferax volcanii* species. The comparative genomics revealed an open pangenome for both genera investigated here. Whole-genome sequence analysis revealed that these isolates have multiple copies of industrially/biotechnologically important unique genes and enzymes. Among these unique enzymes, for recombinant expression and purification, we selected four putative α-amylases identified in these three isolates. We successfully purified functional halotolerant recombinant Amy2, from wsp1 using pelB signal sequence-based secretion strategy using *Escherichia coli* as an expression host. This method may prove useful to produce functional haloarchaeal secretory recombinant proteins suitable for commercial or research applications. Biochemical analysis of Amy2 suggests the halotolerant nature of the enzyme having maximum enzymatic activity observed at 1 M NaCl. We also report the isolation and characterization of carotenoids purified from these isolates. This study highlights the presence of several industrially important enzymes in the haloarchaeal strains which may potentially have improved features like stability and salt tolerance suitable for industrial applications.

## Introduction

The extremely halophilic archaea have adapted to bloom even in harsh environmental conditions such as high salinity, desiccation, and intense solar radiations (Mormile et al., 2003; Gunde-Cimerman et al., 2005; Schubert et al., 2010; Stan-Lotter and Fendrihan, 2015; Winters et al., 2015). These microorganisms require at least 1.5–2.5 M NaCl concentration for their viability and typically grow optimally in 3.5 M NaCl concentrations (Ollivier et al., 1994). Haloarchaea commonly resides in hypersaline environments such as salt lakes, salterns, heavily salted hides, meats, fish, and sauces (Radax et al., 2001; Gruber et al., 2004; Stan-Lotter and Fendrihan, 2015). Adaptation in such extreme and diverse environments makes their genome highly rich in multiple essential genes that are absent in other microorganisms (Papke et al., 2015). This essential new haloarchaeal gene pool analysis has the potential to uncover many industrially important proteins and enzymes. So, it is imperative to perform pangenome or comparative genomics analysis to understand the genetic evolution and distribution of unique and conserved genes in these microbes that help them to survive in harsh conditions (Kim et al., 2018). As aerophilic mesophiles, many haloarchaea are easy to grow in the laboratory conditions, making them one of the most extensively studied archaeal groups and thus, leading to the development of a variety of biochemical, genetic and genomic tools for better understanding of several diverse haloarchaeal species (Soppa, 2006).

Besides having the ability to thrive in high salt conditions, haloarchaea possess diverse physiologies including alkaliphiles, facultative thermophiles, thermoalkaliphiles, and psychrotolerant species (Bowers et al., 2009; Bowers and Wiegel, 2011) and diverse metabolic strategies. These interesting features make them ideal organisms for understanding archaeal biology (Falb et al., 2008). In addition, the genetic basis of these microbes to flourish in hypersaline environments may provide crucial insights to develop salt-tolerant plants for growth in currently non-arable land (Flowers and Colmer, 2015).

Halophiles produce a range of unique and stable biomolecules of commercial applications including (1) hydrolytic enzymes like gelatinases, proteases, lipases, DNAases, xylanases, and amylase. Exo-enzymes from these organisms with polymer degrading ability is of great interest in many industrial processes where high salt concentration would cause enzymatic inhibition (Oren, 2010). These unique features make haloarchaeal enzymes very useful in commercial industries such as baking industries (Oren, 2010), starch liquefaction (Chi et al., 2010), detergent industries, maltose production, etc. (Singh and Singh, 2017). (2) Membrane proteins such as bacteriorhodopsin, commercially recognized for its use in artificial retina, holograms, photoelectrical devices, optical computing, etc. (3) Biodegradable polymers such as polyhydroxyalkanoates (PHAs), produced by many haloarchaea can be used as an alternative to non-degradable plastics (Schiraldi et al., 2002; Oren, 2010). (4) Carotenoids: under certain growth conditions, microbial cells are known to accumulate different pigments, having several commercial applications (Britton, 1995; Vershinin, 1999). Carotenoids are one such class of pigments produced by microbes and plants which play a major role in protecting cells against photo-oxidative damage and hence have vital applications in the environment (Zhang et al., 2014), food and nutrition (Vilchez et al., 2011), disease control (Fiedor and Burda, 2014), and as potent antimicrobial agent (Narsing Rao et al., 2017). Haloarchaea are one of the richest sources of carotenoids compared to other microorganisms (Yatsunami et al., 2014; Montero-Lobato et al., 2018; Giani et al., 2019). The main component of the haloarchaeal carotenoid pool is bacterioruberin which reportedly has more antioxidant properties compared to plant β-carotenes (Yatsunami et al., 2014). Other than bacterioruberin, they also contain isopentenyl dehydrodopin, lycopene, and phytoene in trace amounts (Yatsunami et al., 2014).

In the present study, we isolated and characterized three haloarchaeal strains namely wsp1, wsp3, and wsp4 from the Pondicherry solar lakes, one of the high salt containing areas of the Indian solar salterns. We performed a polyphasic taxonomic classification of these isolated strains using whole-genome sequencing and biochemical assays. Using comparative genomic analysis, we identified the core, accessory, and unique gene pool of haloarchaeal proteins. We identified several industrially important enzymes encoded in the genomes of these strains. We report the expression, and purification of a halotolerant recombinant starch degrading enzyme i.e., α-amylase Amy2 isolated from *Haloferax* strain wsp1 having distinct sequence and structural features. All the strains reported here were colored and genome analysis suggested the presence of carotene biosynthesis genes in their genomes. We further report the isolation and characterization of carotenoids produced by these strains.

## Results

### Isolation and Taxonomic Characterization of Haloarchaeal Isolates

**FIGURE 1:**
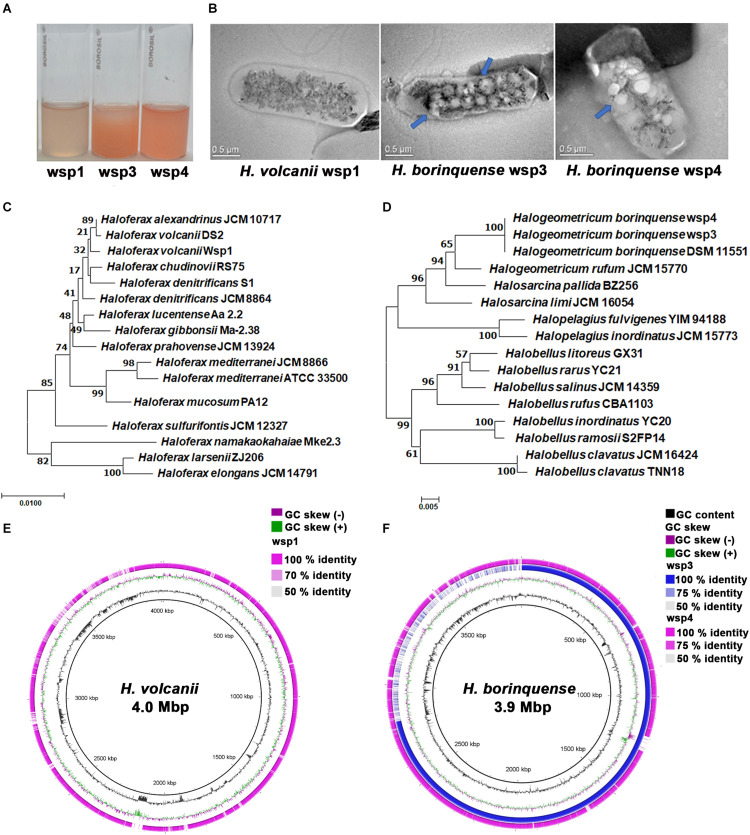
Morphological and taxonomic characterization of the haloarchaeal isolates. **(A)** Wild type culture of haloarchaeal isolates in light reddish-pink color suggesting high carotenoid production. **(B)** TEM images suggest that wsp3 and wsp4 are highly vacuolated (shown in blue arrow) compared with wsp1 **(C,D)** The16S rRNA based phylogenetic analysis suggested that wsp1 strain is closely related to *H. volcanii* DS2 while both wsp3 and wsp4 are closely related to *H. borinquense* DSM 11551. **(E,F)** Genomic maps of the isolates and their reference species constructed using the BRIG tool. The inner black rings show the coordinates in scale and total genome size of the reference sequence, with black histogram representing GC content and purple/green histograms representing GC deviations. In the panel, **(E)** outermost ring in magenta color represents the genome map of wsp1 strain and in the panel, **(F)** blue color represents wsp3 genome map while wsp4 genome map is represented in magenta color.

All three strains were isolated from solar saltern samples collected from Marakkanam solar salterns, India (12°11′13.0272″N and 79°55′40.4220″E) using the dilution-plate technique on the *Halovibrio* agar medium as described previously (Verma et al., 2019). Briefly, on agar plates, the reddish-pink opaque convex colonies, 1–2 mm in diameter appeared in 7 days. The growth of wsp isolates were screened from 12 to 42°C, which suggested that they have optimum growth at 37°C. The positive growth was observed on media containing 3–5 M NaCl (Figure 1A), whereas no growth was observed on 0.3–2 M NaCl concentrations. Transmission electron microscopic images suggested that wsp1, wsp3, and wsp4 have pleomorphic morphology, and compared to wsp1, both wsp3, and wsp4 isolates are highly vacuolated (Figure 1B and Supplementary Figure S1).

### Comparative Genome Analyses of wsp1, wsp3, and wsp4

Comparison of both 16S rRNA and whole-genome sequences suggested that all three isolates are novel strains that belong to *Haloferax* and *Halogeometricum* genera. These results were further confirmed by calculating ANI (Average nucleotide index) and digital DNA-DNA hybridization scores where the observed values were higher than the accepted cut-off values (for ANI > 95% and for DNA–DNA hybridization >91%) for novel species (Supplementary Tables S1, S2). The phylogenetic analysis based on 16S rRNA sequencing data suggested that wsp1 is closely related to *Haloferax volcanii* DS2 and *Haloferax chudinovii* RS75 strains sharing 99% sequence similarity. Both wsp3 and wsp4 are closely related to *Halogeometricum borinquense* DSM 11551 strain sharing >99% sequence identity (Figures 1C,D). The genus *Haloferax* was first described by Torreblanca et al. (1986) and currently comprises twelve species, namely *H. sulfurifontis, H. mucosum, H. mediterranei, H. denitrificans, H. prahovense, H. larsenii, H. gibbonsii, H. elongans, H. alexandrinus, H. lucentense, H. volcanii*, and *H. massiliensis.* On the other hand, genus *Halogeometricum* was first described by Montalvo-Rodriguez et al. (1998) and comprises four species, namely *H. pallidum, H. borinquense, H. limi*, and *H. rufum. H. volcanii* DS2, and *H. borenquencis* DSM 11551 are the type strains of *Haloferax* and *Halogeometricum* genus, respectively. The draft genome sequences of wsp1, wsp3, and wsp4 comprized of 37, 07,582 bp (3.7 Mb), 28, 18,554 bp (2.8 Mb) and 39, 97,080 bp (3.9 Mb) with 4047, 3040, and 4257 annotated coding sequences (CDS). The GC content of wsp1, wsp3, and wsp4 were 66.1, 61.0, and 59.7%, respectively. For further analysis, we compared wsp1 genome sequences with *H. volcanii* DS2 and both wsp3 and wsp4 genomes with the *H. borinquense* DSM 11551. Genome circular map built by using BLAST Ring Image Generator (BRIG) tool (Alikhan et al., 2011), revealed that wsp3 has high sequence similarity with both wsp4 and *H. borinquense* DSM 11551 genomes except one highly variable region of about 1 Mbp (Figure 1F).

### Pangenome Analysis

During evolution, microbes acquired several genes that facilitate their growth and survival. The important genes in this list include genes responsible for cell signaling, metabolic regulators, antibacterial proteins, ion transporters, etc. To understand the environmental effect on gene pool variation, we performed a pangenome analysis of wsp samples with their closely related species using the Bacterial Pan Genome Analysis (BPGA) pipeline (Chaudhari et al., 2016). The original pangenome concept was developed by Tettelin et al. (2005) and it describes the total pool of genetic material comprized of all members of a species. The pangenome consists of three different groups known as the core, accessory, and unique genomes. The core genome consists of common genes that are present in all individuals, accessory or dispensable genome containing shell genes present in few individuals and a unique genome contains genes that are specifically present only in an individual member (Tettelin et al., 2005; Wolf et al., 2012; Vernikos et al., 2015). The outcomes of the pangenome analysis are discussed in the following sections.

#### Clusters of Orthologous Groups (COG) Distribution Plots

**FIGURE 2:**
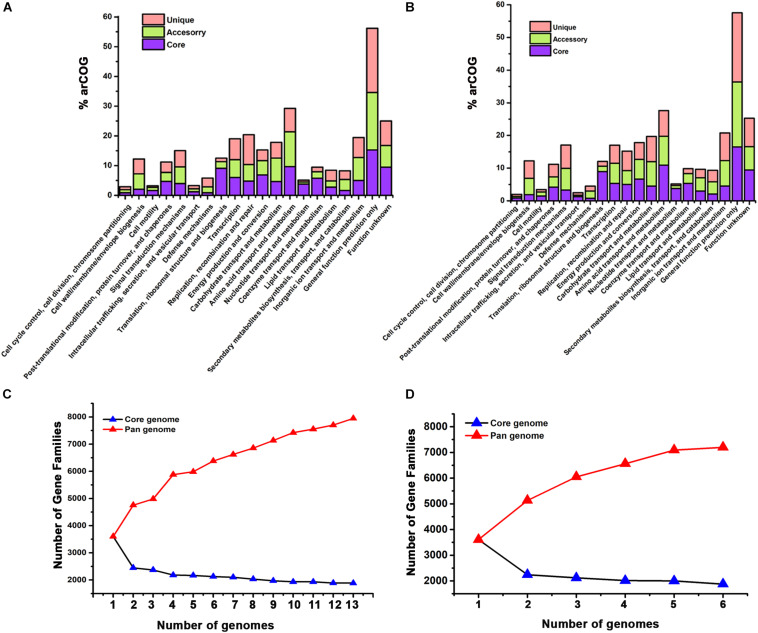
Archaeal clusters of orthologous groups (arCOG) analysis of the genomes. Comparison of the COG distribution between the core, accessory and unique genes of the **(A)** thirteen species belonging to genus *Haloferax* including wsp1 and **(B)** four species belonging to genus *Halogeometricum* including wsp3 and wsp4 have been analyzed using default parameters of BPGA pipeline. The arCOG categories are indicated on the *X*-axis and the percentage of the genes present in each category of the arCOG classes is indicated on the *Y*-axis. **(C,D)** The Core and pangenome plots of genera *Haloferax* and *Halogeometricum*, respectively. Red and blue colored lines indicate the change in the number of pangenome and core genome gene groups added sequentially to the genome.

The whole-genome sequences of *Haloferax* and *Halogeometricum* members were retrieved from NCBI (National Center for Biotechnology Information) database. The functional annotation was carried out using Rapid Annotation using Subsystem Technology webserver (Aziz et al., 2008). The distribution of the archaeal clusters of orthologous groups (arCOG) of *Haloferax* and *Halogeometricum* genera along with our isolates with their biological functions are shown in Figures 2A,B. The potential functions of the unique genes from both genera appear to be widespread and linked with many different cellular functions such as cell motility, post-translational modifications, chaperones, signal transduction mechanisms, and many genes with unknown function. On the other hand, the accessory genes identified are mainly associated with carbohydrate transport, inorganic ion transport, and several genes with unknown function. The core genome consists of the genes involved in translation regulation, ribosomal structure and biogenesis, replication, recombination and repair proteins, energy production and conversion, coenzyme transport and metabolism, lipid transport and metabolism and nucleotide transport and metabolism.

#### Pan and Core Genome Plots

The comparative genome analysis revealed an open pangenome for both genera investigated here (Figures 2C,D) as the number of accessory and unique genes are increasing with the addition of new genomes. For the genus *Haloferax*, the pangenome and core genome contain 7950 and 1885 genes, respectively (Figure 2C). For the genus *Halogeometricum*, the pangenome and core genome contain 7197 and 1874 genes, respectively (Figure 2D). Similar findings were reported for the pangenome based analysis in other studies as well (Capes et al., 2012; Becker et al., 2014; Borchert et al., 2017). The pangenome analysis revealed that wsp1, wsp3, and wsp4 contain 246, 530, and 106 unique genes in their genomes. These unique genes were further annotated using online server Web Services for Metagenomic Analysis (WEBMGA) (Wu et al., 2011). We found multiple unique clusters including genes potentially encoding cation efflux proteins, integrases, and proteins potentially involved in multidrug resistance. The annotated genes also included commercially useful salt-tolerant enzymes like amylases, alcohol dehydrogenases, sulphataes, esterase’s, etc.

The size of pangenome is larger than the core genome and is increasing with the addition of the new genomes suggesting both the genera investigated here have open pangenome. The current study is important for understanding the genomic variations and distribution of accessory genes providing survival advantage to the haloarchaeal strains in adverse conditions.

### Biochemical Characterization of Haloarchaeal Isolates

Polyphasic taxonomic characterization were studied according to the methods in the proposed minimal standards for description of new taxa in the order *Halobacteriales* (Table 1A; Grant and Larsen, 1989; Oren et al., 1997). Biochemical characterization suggested that all three isolates were positive for the catalase test and negative for urease, lipase, and Voges-Proskauer tests. All three strains were also negative for acid production, casein hydrolysis, and hydrogen sulfide production. We also performed enzymatic screening which involved in amino-acid biosynthesis pathways and found that all three were negative for lysine decarboxylase, arginine decarboxylase, aesulin hydrolysis, and ornithine decarboxylase.

**TABLE 1:** **(A)** Biochemical test and **(B)** carbon source utilization test as an aid to the classification of haloarchaea.

	Wsp3	Wsp4	Wsp1
**(A) Biochemical test**			
Catalase test	+	+	+
Oxidase Test	+	+	−
Citrate utilization	+	+	−
Production of acid	−	−	−
Production of gas	−	−	−
Lysine decarboxylase	−	−	−
Methyl red test	+	+	−
Voges-Proskauer test	−	−	−
Nitrate reduction	−	+	+
Casein hydrolysis	−	−	−
Arginine decarboxylase	−	−	−
Urease activity	−	−	−
Ornithine decarboxylase	−	−	−
Aesulin hydrolysis	+	+	+
Gelatin Hydrolysis	+	−	−
Indole production	+	−	−
Lipase production	−	−	−
**(B) Carbon source**			
Dextrose	W	W	W
Pyruvate	W	W	−
Lactose	−	−	+
Maltose	+++	W	−
Sucrose	W	−	−
Galactose	−	−	+++
Arabinose	−	−	−
Rhamanose	−	−	−
Cellobiose	W	−	−
Aldonitol	−	−	−
Inositol	−	−	−
Inulin	−	−	−
Dulcitol	W	−	−
Mannose	−	−	−
Melibiose	−	−	−

*+++, highly positive; +, positive; **−**, negative, W, weakly positive.*

Wsp3 and wsp4 strains had many similar enzymatic features such as both were positive for citrate utilization, oxidase test, and methyl red test. Besides these similarities, some variations were also observed e.g., both *H. borinquense* DSM 11551 and wsp3 are positive for gelatin liquefaction and indole production while wsp4 is negative. We also found that wsp3 is unable to reduce nitrate, unlike *H. borinquense* DSM 11551 and wsp4. Genome analysis suggests that both *H. borinquense* DSM 11551 and wsp4 encode nitrate reductase gene which is missing in the wsp3 genome. Similarly, we also found a copy of the nitrate reductase gene in wsp1 genome, which reduces nitrate, whereas it is missing in *H. volcanii* DS2 which is negative for nitrate utilization.

### Carbon Source Utilization

Carbon source preferences of haloarchaeal isolates were identified by monitoring their growth in the presence of different carbon sources. Optical density-based assay results suggest that all three strains have similar preferences for dextrose while they were unable to utilize arabinose, rhamnose, aldonitol, inositol, inulin, and melibiose (Table 1B). Along with enzyme-based biochemical tests, wsp3 and wsp4 had some similar and dissimilar preferences for carbon source utilization. Both wsp3 and wsp4 were positive for pyruvate and maltose utilization while negative for lactose and galactose utilization. Surprisingly, wsp3 alone was positive for three different carbon sources i.e., sucrose, cellobiose, and dulicitol while both *H. borinquense* DSM 11551 and wsp4 were negative. Similarly, wsp1 is not able to utilize sucrose and arabinose while its type strain *H. volcanii* DS2 is capable of utilizing both sugars.

Quantitative analysis of carbon source utilization shows that both wsp1 and wsp3 efficiently utilize galactose and maltose, respectively. Genome analysis suggested that both wsp1 and *H. volcanii* DS2, encode galactokinase enzyme which help them to utilize galactose as a carbon source (Anderson et al., 2011) while this enzyme is absent in both wsp3 and wsp4 strains. For maltose utilization, two alternative pathways have been proposed (Cibrario et al., 2016). The two essential enzymes of these pathways are *malz* (α-1, 4-glucosidase) and *malA* (α-amylase MalA). Genomic data analysis suggested that only wsp3 genome encodes for two different copies of α-1, 4-glucosidase enzyme while wsp1 and wsp4 only have an α-amylase gene in their genomes. This probably explains why wsp3 can utilize maltose more efficiently compared to both wsp1 and wsp4.

### Antibiotic Susceptibility Profile

Culture isolates were also screened for antibiotic resistance (Table 2). The analysis suggested that all three isolates were sensitive for novobiocin and resistant to penicillin, vancomycin, chloramphenicol, cephadroxil, lincomycin, cephalexin, ceftazidine, and cephadroxil. Surprisingly wsp3 had sensitivity for cefazolin and kanamycin while wsp1 and wsp4 were resistant to both the antibiotics like their type strains. The possible reason for kanamycin resistance in wsp1 and wsp4 is the presence of aminoglycoside phosphotransferase while this enzyme is absent in wsp3 hence probably explaining the differences observed in kanamycin resistance in these isolates.

**TABLE 2:** Antibiotic susceptibility test of the isolates.

Antibiotic sensitivity	wsp3	wsp4	wsp1
Novobiocin	S	S	S
Cefazolin	S	R	R
Kanamycin	S	R	R
Bacitracin	I	R	R
Penicillin	R	R	R
Vancomycin	R	R	R
Chloramphenicol	R	R	R
Cephadroxil	R	R	R
Lincomycin	R	R	R
Ciphalexin	R	R	R
Ceftazidine	R	R	R
Cephadroxil	R	R	R

*S, sensitive; R, resistance; I, intermediate.*

### Carotenoid Isolation and Spectroscopic Characterization

**FIGURE 3:**
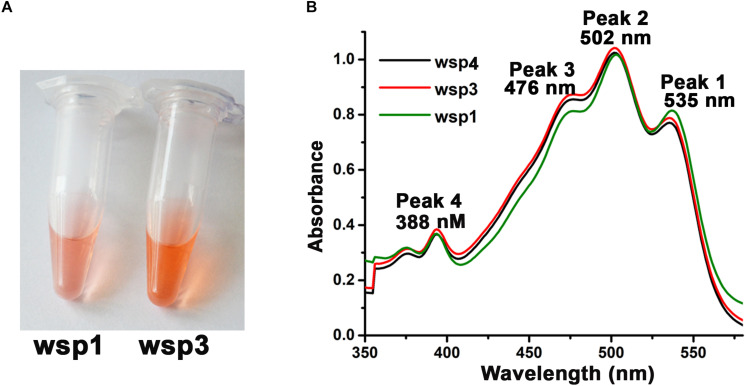
Isolation and biophysical characterization of haloarchaeal carotenoids isolated from wsp1, wsp3, and wsp4 isolates. **(A)** The microcentrifuge tubes containing purified carotenoids from equal culture volume of the two haloarchaeal strains showing observable color variations. **(B)** UV–visible absorption spectra of the purified carotenoids showing four carotenoid standard peaks (peak I–IV) at 535, 502, 476, and 388 nm.

In the present manuscript, we isolated carotenoids from three haloarchaeal strains (Figures 3A,B). The visible cell pellet color of wsp1 was different when compared to both wsp3, and wsp4 suggesting either variations in the content or molecular structure of the carotenoids produced by these strains (Figure 3A). For further analysis, we purified carotenoids following Yatsunami et al. (2014) protocol and characterized them using UV–visible spectroscopy. UV–visible spectra suggested that wsp1, wsp3, and wsp4 have a similar absorption spectrum having major absorption peaks at 476, 502, and 535 nm, however, there were differences in the peak heights (Figure 3B). These differences may explain why carotenoids isolated from wsp1 are different in color compared to wsp3 and wsp4. These three major peaks correspond to bacterioruberin (535 nm), all-Trans-lycopene (502 nm), and 13-cis-lycopene (476 nm). Bacterioruberin is a major component of haloarchaeal carotenoids which is synthesized from lycopene (Yang et al., 2015). We took the ratio of peak1/peak3 which may help us understand the bacterioruberin and lycopene contents in these strains. The lycopene has specific absorption at 502 and 476 nm while bacterioruberin absorbs at 535 nm. A slight absorption of bacterioruberin is also reported at 466 nm. The peak1/peak3 ratio was 1.0, 0.9, and 0.8 for wsp1, wsp3, and wsp4, respectively, suggesting that wsp1 has the highest bacterioruberin content among the three strains.

### Recombinant Expression and Purification of Halotolerant α-Amylase From the Isolates

Haloarchaea lives in hypersaline environments such as marine salterns, saline soils, soda lakes, salted foods, etc. The cell lysis of marine planktons such as shrimps, diatoms, algae, fish, purple and green bacteria, etc. release different biomolecules including starch, cellulose, proteins, chitin, etc. To digest this biopolymer, haloarchaea needs to produce different digestive enzymes to utilize these substrates.

The whole-genome sequencing and annotation results suggested that wsp1, wsp3, wsp4 genomes code for two, one, and three putative α-amylase genes, respectively. We successfully cloned all four α-amylase genes sharing <40% sequence identity among themselves. However, only one of them, named *amy2* (locus tag G3A49-11660) isolated from wsp1, expressed well. Multiple domain analysis of Amy2 (protein ID QIB80089.1) suggested that it has a conserved α-amylase domain in the C-terminal region (residue range 227 to 635). A PSI-BLAST search using the NCBI database was performed for the additional N-terminal region (residue ranges from 1 to 226 aa). However, we did not find any significant hits, therefore, the function of this N-terminal domain is still not clear. Initially, we attempted to purify recombinant His-tagged Amy2 from the cytosolic fraction using *E. coli* Rosetta DE3 as an expression host. We successfully purified Amy2, however, the purified enzyme was inactive as suggested by the starch agar plate assay. Since most of the amylases are secretory proteins so we cloned *amy2* in pET22b vector having pelB secretion signal sequence at the N-terminus to aid secretion/expression in the periplasmic space and C-terminal 6X His-tag to aid purification of the recombinant Amy2. We used this construct for purification and further biochemical characterization of Amy2.

### Biochemical Characterization of Amy2

**FIGURE 4:**
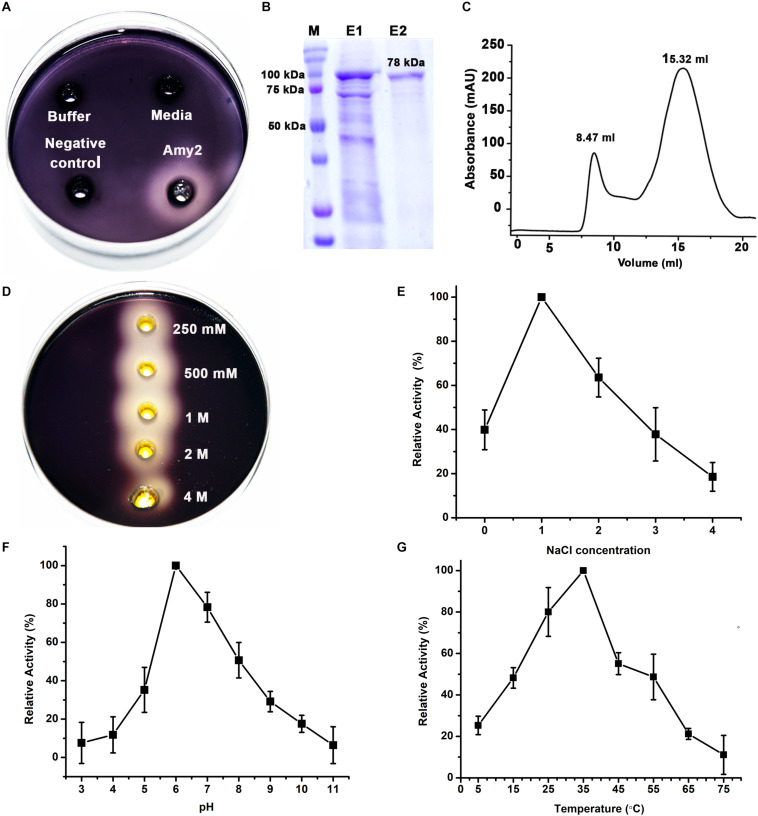
The purification of recombinant α-amylase from *E. coli*, and carotenoids from wild type cultures. **(A)** The α-amylase activity observed from the cell-free supernatant of *E. coli* expressing recombinant Amy2. **(B)** The 15 % SDS-PAGE showing a predominant protein band at the expected molecular weight of ∼78 kDa in the gradient of imidazole elution fractions (E1, E2, E3 correspond to 20, 100, and 200 mM imidazole, respectively). **(C)** The analytical gel filtration profile suggests that Amy2 is predominantly monomeric in solution. A small fraction, corresponding to large aggregates, was also observed in the void volume (8.47 ml). **(D)** Evaluating salt tolerance of Amy2 using starch plate assay suggests that the enzyme is halotolerant. **(E)** The enzymatic activity of Amy2 against soluble starch was assayed by the iodine-starch method at the various concentrations of NaCl. Relative activity was defined as the percentage of maximum activity at 1 M NaCl. Plots showing effect of pH **(F)** and temperature **(G)** on the Activity of Amy2 and the relative activity was defined as the percentage of maximum activity observed at pH 6.0 or 35°C, respectively. All activity assays were performed three times in triplicate and mean value with standard deviation are plotted.

Upon induction, we observed amylase activity even in the cell-free culture supernatant (Figure 4A). *E. coli* cells alone or cells carrying the pET-22b vector without amylase genes were used as negative controls in these experiments (Figure 4A). We successfully purified Amy2 from the periplasmic space using Ni-NTA-based affinity chromatography (Figure 4B). The gel-filtration profile suggested that Amy2 is predominantly monomeric in solution (Figure 4C) and enzymatically active on the starch agar plates. We initially tested Amy2 salt tolerance by incubating purified enzyme for 12 h with different concentrations of salt ranging from 0.25 to 4 M on the starch agar plate (Figure 4D). We observed amylase activity in all the samples suggesting halotolerant property of Amy2. We further performed the biochemical characterization of Amy2 to study its stability and activity at various salt concentrations, pH, and temperature.

#### Effect of Salt Concentration on Enzyme Activity

The effect of NaCl concentration ranging from 0–4 M on enzyme activity was examined. Data suggests that Amy2 is active in all the NaCl concentrations studied having maximum activity at 1 M NaCl concentration. These results suggest that Amy2 has wide-range salt tolerance and retains moderate activity even at 3 M NaCl concentrations (Figure 4E).

#### Effect of pH on Enzyme Activity

The effect of pH on activity was observed by performing enzyme assay under pH ranging from 3.0 to 11.0 at 1unit interval for 30 min at 37°C. The maximum enzymatic activity was observed at pH 6.0, while ∼30% activity was observed at pH 5.0 and pH 9.0 suggesting enzyme is active at wide pH range (Figure 4F).

### Effect of Temperature on Enzyme Activity

The thermal effect on enzymatic activity was studied at the temperature range of 5–75°C. Our finding revealed that Amy2 showed maximum activity at 35°C, and retained 40% activity at 55°C whereas negligible activity was observed at 75°C (Figure 4G).

The biochemical characterization reveals attractive features of Amy2 including halotolerant, moderately thermostable, and activity at a wide pH range suitable for various industrial applications. These biochemical characteristics reported here are comparable to α-amylases reported from other halophiles (Onodera et al., 2013; Bajpai et al., 2015).

### Structure Prediction and Analysis of Amy2

Most of the archaeal α-amylases belong to the subfamily of glycosyl hydrolase GH families: GH13, GH70, and GH77 (Dong et al., 1997; Bowers et al., 2009). In recent years, several potential α-amylases from different halophilic archaea have also been added to these families (Lombard et al., 2014; Santorelli et al., 2016). The enzyme belonging to this families should have three distinct domains: a central catalytic domain harboring a (β/α)_8_ Tim barrel (domain A), with an irregular loop domain (domain B) usually protruding as a long loop out of the barrel connecting the third β-strand and the third α-helix and with the typical structure of an antiparallel β-sandwich (domain C) (Sarian et al., 2017).

**FIGURE 5:**
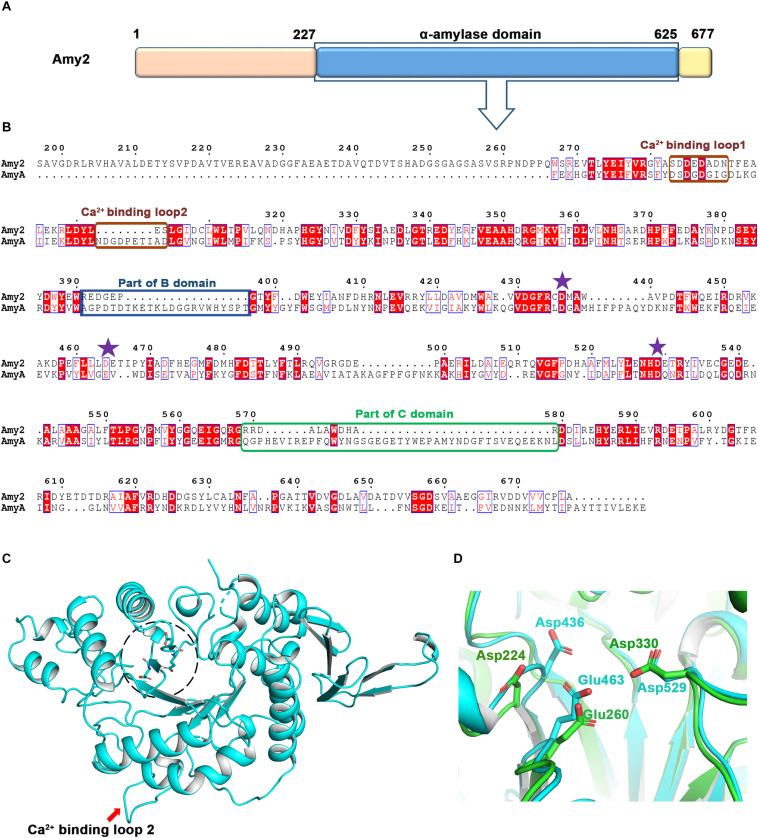
Structural and sequence features in Amy2. **(A)** Schematic representation of predicted domain architecture in Amy2. The N-terminal (1–226) region, present in α-amylases from other haloarchaeal strains as well, did not show significant sequence similarity with proteins of known function. **(B)** Multiple sequence alignment of the Amy2 region sharing sequence conservation with *H. orenii*αα-amylase. The active site residues are marked in purple stars. The differences in the Ca^2+^ binding loops, and domains B and C are highlighted in colored boxes. **(C)** Predicted structural model of Amy2 built using the PHYRE2 online web server. The active site is encircled in black broken circle and active site residues are shown in stick representation. **(D)** The structural alignment suggested that Amy2 (cyan) and *H. orenii* α-amylase (green) have conserved active site triad consisting of structurally equivalent Asp436, Glu463, and Asp529 residues.

Multiple sequence alignment using the NCBI blast portal revealed that Amy2 has a catalytic α-amylase domain encompassing 267–600 residue range (Figure 5A). This catalytic domain is mainly present in archaeal and bacterial species and is known to hydrolyze α-(1, 4) glycosidic linkages of glycogen, starch, related polysaccharides, and some oligosaccharides (Antranikian, 1992; Gupta et al., 2003; Sivaramakrishnan et al., 2006). The structural model of the catalytic domain of Amy2 (R269-D625) was built using PHYRE2 (Kelley et al., 2015) webserver suggested that it may have a similar structural architecture found in other members of GH family of amylases i.e., eight stranded alpha/beta-barrel that contains the active site, calcium-binding domain present between beta-strand 3rd and alpha-helix 3rd, and a carboxyl-terminal Greek key beta-barrel domain (Abe et al., 2005). Amy2 sequence blast at the RCSB database (Bernstein et al., 1977) suggested that *Halothermothrix orenii* α-amylase is the closest structural homolog sharing 28% sequence identity over 426 residues (ranges from 3 to 429 aa) (Figure 5B). The majority of the α-amylase enzymes are calcium-dependent metalloenzymes, where metal ion is required for both stability and enzymatic activity (Hsiu et al., 1964; Boel et al., 1990). *H. orenii* α-amylase (PDB ID-1WZA) harbors two different calcium-binding loops, loop 1: Asp44 aa to Ile52 aa and loop 2: Asp65 to Asp73 residues (Sivakumar et al., 2006). The sequence alignment and predicted structural model suggests that the calcium-binding loop 2 is missing in Amy2 (Figures 5B,C). Amy2 also has smaller B and C domains compared to *H. orenii* α-amylase due to deletions in the loop regions (Figures 5B,C).

For amylase activity, a catalytic triad consisting of Asp-Glu-Asp is required where first aspartic acid is involved in attacking the sugar anomeric center by a nucleophilic side chain and this reaction is assisted and governed by other two Glu-Asp residues (Hsiu et al., 1964; Machius et al., 1995). In *H. orenii* this triad is composed of Asp224-Glu260-Asp330. Multiple sequence alignment-based analysis suggests that Amy2 also has similar tried consisting of Asp436-Glu463-Asp529, respectively (Figure 5D).

### Screening of CRISPR Sequences and Secondary Metabolites

The clustered regularly interspaced short palindromic repeats (CRISPR)-Cas system is used by bacteria and archaea to fend off foreign genetic elements (Horvath and Barrangou, 2010). Since its discovery, it has been developed into numerous applications like genome editing and regulation of transcription in eukaryotes and bacteria (Gilbert et al., 2013). The genomes of the strains wsp1, wsp3, and wsp4 were submitted to CRISPR Finder^1^ (Grissa et al., 2007) which suggested that all three genomes have multiple CRISPR-DR (CRISPR-direct repeat) as well as spacer sequences. In wsp1, wsp3 and wsp4 genomes six, one, and four CRISPR-DR sequences were observed, respectively (Supplementary Table S3). Whole-genome data of wsp1, wsp3, and wsp4 were further analyzed for the presence of CRISPR associated genes (Cas). Our analysis suggested the presence of several Cas sequences in both wsp1 and wsp4 genomes although no Cas-related genes were identified in the wsp3 genome. In haloarchaea CRISPR-Cas system type I, subtype I-B, is dominantly present (Maier et al., 2017). A characteristic feature of type I systems is the presence of nuclease Cas3 protein, and subtype I-B is further characterized by the presence of Cas8b (Makarova and Koonin, 2015). The functional process of Type I-B system is mainly based on the presence of Cas proteins, namely, Cas5, Cas7, and Cas8b which combine to form a Cascade (CRISPR-associated complex for antiviral defense)-like complex in different *Haloferax* species such as *H. volcanii* (Maier et al., 2019). Genome analysis of both wsp1 and wsp4 suggested that they encode all the genes required to type I CRISPR system i.e., Cas1, Cas2, Cas3, Cas4, Cas5, Cas6, and Cas8b genes. The presence of both Cas3 and Cas8b suggests that pws1 and pws4 harbor CRISPR-cas type IB system similar to other haloarchaeal species.

In addition to enzymes, recent studies indicate that archaea can also produce a wide range of small peptides and secondary metabolites (Pietra, 1997; Pettit, 2011; Wang et al., 2019). These small peptides and secondary metabolites could be of considerable interest in biotechnology. For the screening of secondary metabolite production, all three genome sequences were uploaded onto an online antiSMASH server (Medema et al., 2011). The output results predicted multiple copies of two important secondary metabolites i.e., siderophore and terpenes in all three strains (Supplementary Table S4). In the case of wsp3, no siderophore gene was predicted. Siderophores play an important role in iron transport (Neilands, 1995) while the functions of terpenes in haloarchaea are not known yet.

## Discussion

Haloarchaeal genera show huge variations in the diversity within different geographical locations (Oren, 2002; Naghoni et al., 2017). There are several reports which suggest that different haloarchaeal strains were routinely isolated from the different salt areas like solar salterns, brines, salt lakes, salt pans, rock salts, etc. (Oren, 2002; Javor, 2012). In the present study, three haloarchaeal strains, namely wsp1, wsp3, and wsp4, were isolated from the high salinity environment and studied in detail. Haloarchaeal isolates adopt diverse morphological shapes like rods, pleomorphic rods, square, cocci, triangles, and disks (Burns et al., 2007; Minegishi et al., 2010). Few haloarchaeal strains show rod form morphology in liquid culture while they are motile and formed nonmotile cocci on solid agar medium (Grant, 2001). All of the three isolates in this study i.e., wsp1, wsp3, and wsp4 were highly pleomorphic. Also, wsp3 and wsp4 were highly vacuolated, which may help these microbes to float on the surface of the water (Oren, 2012).

The pangenome comparison of the four *Halogeometricum* and twelve *Haloferax* haloarchaeal genomes, including three isolates reported in this study, have revealed genomic variations resulting from horizontal gene transfer, gene duplication, gene loss events, etc. which is in line with results from other studies (Vos and Didelot, 2009; DeMaere et al., 2013). This data also suggests that pangenomes of these genera are open and each genome contains about 3 to 20% unique genes. The pangenome analysis further suggested that the distribution of the unique, accessory, and core genes is widespread across different biological functions and no obvious pattern was observed.

Our data and past studies (Nagata et al., 1989; Littlechild, 2015; Karray et al., 2018; Amoozegar et al., 2019) suggest that haloarchaea codes for several enzymes suitable for industrial and research applications. Especially, their salt-tolerant and thermostable nature are attractive features suitable for specific applications like food processing, biofuel production, detergent additives, etc. However, the potential of these enzymes has not yet been commercially exploited. Amylase enzymes are typically involved in essential processes in microorganisms, such as hydrolysis of starch and glycogen, yielding glucose and maltose (Werries and Muller, 1986). There is an increased interest in exploiting amylases for the conversion of grain starch into fermentable sugars for ethanol production (Shigechi et al., 2004; Prakash and Jaiswal, 2010). However, purification of enzymes from the natural haloarchaeal source will not be commercially viable owing to the poor yield and slow growth rate (doubling time ∼4–10 h). Hence, there is a need for developing processes based on the recombinant expression of the target enzymes using fast-growing microbial hosts like *E. coli*. There are limited studies that report the recombinant haloarchaeal α-amylase production (Perez-Pomares et al., 2003; Hutcheon et al., 2005; Bajpai et al., 2015; Santorelli et al., 2016). Onodera et al., produced recombinant *Haloarcula japonica* α-amylase using *E. coli* host and found that malA is a cytosolic halotolerant α-amylase which is active even at 4 M salt concentration (Onodera et al., 2013). Our whole-genome data analysis of the wsp isolates suggested that they all code for one or more types of α-amylase genes in their genomes. We were able to successfully clone, express, and purify one of the annotated α-amylase genes named *amy2* from wsp1. Interestingly, though soluble, Amy2 purified from the cytosolic soluble fraction was not active. α-amylases are usually secretory proteins and there are five cysteine residues in Amy2. So, to direct the protein to the periplasmic space for proper folding and functional expression, we cloned Amy2 having pelB signal sequence at the N-terminus. Using this strategy, we were successful in purifying active Amy2. In future, this strategy may aid purification of other functional α-amylases as well. The purified Amy2 was studied for its activity in the presence of varying salt concentration, pH, and temperature conditions. Data suggests that Amy2 was active in a wide range of salt concentrations, temperature, and pH conditions. However, detailed studies are required to further investigate its suitability for various industrial applications.

All three isolates were characterized based on a battery of biochemical tests and their antibiotic susceptibility was also evaluated. The results for these assays for wsp1, wsp3, and wsp4 were similar to their corresponding type strains. However, some differences were also observed which we were able to explain based on the whole genome sequencing data analysis. These discrepancies were easily correlated with the presence or absence of the corresponding enzyme(s) in the genome as described in the results section. Haloarchaea is known for producing high levels of carotenoids like bacterioruberin and its C50-related pigments having high commercial potential in various industries including cosmetic, food, poultry and health (Vilchez et al., 2011; Mata-Gomez et al., 2014; Naziri et al., 2014; Rodrigo-Banos et al., 2015). Interestingly, haloarchaeal carotenoid has also been shown to have dose-dependent cytotoxicity against human liver cancer cell lines suggesting anti-cancer activity (Rodrigo-Banos et al., 2015). The isolated carotenoids from all of the three strains in this studies showed a characteristic absorption profile similar to other haloarchaeal strains (Yatsunami et al., 2014; Yang et al., 2015). The differences in the UV–Vis spectrum observed among the carotenoids purified from these strains suggest differences in the contents of bacterioruberin and other carotenoids.

To summarize, we have characterized three haloarchaeal strains isolated from Indian solar salterns. We identified several commercially important enzymes encoded in these three haloarchaeal strains and successfully purified halotolerant recombinant α-amylase using *E. coli* as an expression host. Haloarchaea is known to survive harsh environmental and nutrition depleted conditions. The detailed biochemical and biophysical characterization, in the future, may also help explore the feasibility of these enzymes for commercial applications. The future studies aimed at deciphering the function of hypothetical unique and accessory genes may also help in identifying the mechanisms of stress adaptation that aid survival of haloarchaea in harsh conditions.

## Materials and Methods

### Isolation and Biochemical Characterization of Haloarchaeal Isolates

For all the biochemical tests, respective basal mediums (composition: 250.0 g NaCl, 20.0 g MgSO_4_.7H_2_O, 3.0 g trisodium citrate 2H_2_O, 2.0 g KCl, 10.0 g peptone, 25.0 g agar in 1000 ml; pH 7.2) were supplemented with a final concentration of 25.0% NaCl. The growth of the isolates was tested at 12, 15, 20, 25, 30, 37, and 42°C on Halovibrio agar (1% agar added to basal medium). Isolates were plated on Halovibrio medium (without NaCl) (HiMedia) supplemented with different concentrations of NaCl (2, 5, 7, 10, 12, 15, 18, 20, 22, 25, 27 and 30%) to test salt tolerance. Growth at different pH (5.0, 6.5, 7.0, 8.0, 9.0, and 10.0) was examined using *Halovibrio* medium (without NaCl) supplemented with 25% NaCl as described earlier (Chen et al., 2016). The pH was adjusted using different buffers: MES (pH 5.0–6.0), PIPES (pH 6.5–7.0), HEPES (pH 7.5–8.0), and CHES (pH 9.0–10). Catalase activity, citrate utilization (using Simmons’ citrate agar) and urea hydrolysis were determined as described by Cowan and Steel (Cowan and Steel, 1965). The hydrolysis of casein, gelatin, starch and indole, methyl red test, Voges–Proskauer test and oxidase activity were assessed as described by Smibert and Krieg (Smibert et al., 1994). Nitrate reduction was tested as described by Lanyi (Lányi, 1988). Acid production from various sugars was tested on minimal medium by using the method described by Smith et al. (Sasser, 1990). The utilization of different carbohydrates as sole carbon source was tested in minimal medium supplemented with 25.0 % NaCl and 2% MgSO_4_7H_2_O with 1% different carbon sources.

### The Whole-Genome Sequencing and 16S rRNA Comparison

Genomic DNA extractions were performed using the Zymogen DNA isolation kit (Cat. No. D6105). The 16S rRNA gene sequences of strain wsp1, wsp3, and wsp4 were PCR-amplified using 21 forward (TCCGGTTGATCCYGCCGG) and 1453 reverse (GGGCYGCACGCGYRCTACA) standard primers. The amplified 16S rRNA gene sequences were further aligned with those of representative related taxa using the EzTaxon server (Yoon et al., 2017). The 16S rRNA gene sequences of wsp1, wsp3, and wsp4 representatives closely related species were retrieved from the EzTaxon server (Chun et al., 2007) and aligned using MEGA version X (Tamura et al., 2004; Kumar et al., 2018). Phylogenetic trees were constructed using the neighbor-joining methods (Saitou and Nei, 1987) and the percentage of replicate trees in which the associated taxa clustered together in the bootstrap test (1500 replicates) are shown next to the branches (Felsenstein, 1985). The whole-genome sequencing of all three strains were performed using Illumina NextSeq and for assembly CLC NGS Cell ver 9 (CGWB) were used. The draft genomes were annotated using the RAST online server (Aziz et al., 2008). The *H. borinquense* DSM 11551 and *H. volcanii* DS2 genomes were used as a reference for comparison and annotation. Pangenome analysis was carried out by using the BPGA pipeline (Chaudhari et al., 2016). The genomes were screened for secondary metabolite gene clusters using antiSMASH online server (Medema et al., 2011).

### Genome-Sequence Submission

The genome sequence of wsp1, wsp3, and wsp4 are submitted at NCBI under accession numbers as NZ_CP048738, NZ_CP050274 and NZ_CP048739.

### Transmission Electron Microscopy

Aliquots of 50 μl from samples containing wild type culture (basal medium supplemented with 25.0% NaCl) were pipetted on a carbon-coated 300-mesh copper grid (Polysciences, United States). The excess sample was blotted and followed by air drying. The grids were further imaged using a JEM 2100 electron microscope, operated at 200 keV (JEOL).

### Cloning, Expression and Purification of Amy2

The full length of *amy2* was amplified and cloned in pET22b (Novagen) over-expression vector between NdeI-XhoI and NcoI-XhoI sites to yield recombinant protein having C-terminal 6X His-tag. The first set of restrictions were used for cytosolic expression (NdeI-XhoI) while the other set was used to express Amy2 fused with pelB (NcoI-XhoI) secretion signal sequence at the N-terminal. Forward primers 5′AGCTCATATGGCTGTCGGCGAGTCAGTA and 5′AGTAC CATGGCTGTCGGCGAGTCAGTA having NdeI and NcoI sites, respectively, and reverse primer 5′AGTCCTCGAGGGCGAGC GGGCAGACGAC having XhoI site were used for gene amplification. The amplified PCR products and vector were digested with NdeI-XhoI and NcoI-XhoI sites followed by an overnight ligation at 4°C. The ligated products were transformed into Top10 chemical competent cells (Thermo Scientific, United States). Positive clones were screened using colony PCR method. The positive clones were further confirmed by DNA sequencing. The protein was expressed using BL21-DE3 Rosetta cells (Thermo Scientific, United States). Transformed cells were inoculated into 10 ml media as a primary and then into 1000 ml media supplemented 100 μg/ml ampicillin. The cultures were induced at 0.6 OD_600_ by adding 0.3 mM IPTG and incubated overnight at 18°C. Amy2 was purified using Ni-NTA (Merk-Sigma, United States) based affinity chromatography method following standard manufacturer’s instructions. Cell pellets were dissolved in buffer A (20 mM Tris pH 7.4, 250 mM NaCl and 10 mM CaCl_2_) and lysed by sonication. The sample was centrifuged at 12,000 g for 30 min. After centrifugation, pellet was discarded and the cell free supernatant was mixed with Ni-NTA resin. Protein was eluted by adding increasing the concentration of imidazole (20–500 mM) to the buffer A. The eluted fractions were loaded on 15% SDS PAGE to check purity and quality of the protein samples. The elution fractions were pooled and concentrated using 30 K centrifugal amicon ultrafiltration devices (Merk Millipore, United States). The protein was further purified using gel filtration chromatography using Superdex 200 Increase column. The purified Amy2 was used for further biochemical experiments.

### Analytical Gel Filtration

The 0.5 ml of the purified protein sample was injected into the Superdex 200 Increase (GE Healthcare, United States) analytical gel filtration column pre-equilibrated with 20 mM Tris pH 7.4, 250 mM NaCl and 10 mM CaCl_2_. The flow rate was kept at 0.5 ml per min. The chromatogram was recorded at both 280 and 220 nm.

### Carotenoid Isolation

The carotenoids were extracted by following protocol published by Yatsunami et al. (2014). Briefly, 20 ml of haloarchaeal cultures were centrifuged at 12,000 g for 30 min at 25°C. The supernatant was discarded and pellets were further resuspended in 5 ml of acetone and methanol solution (7:3). This solution was incubated at room temperature for 30 min in dark followed by centrifugation at 12,000 *g* for 30 min. Reddish-pink supernatant was collected and pellets were discarded. Isolated colored supernatants were centrifuged at 3000 g in the speed-vac (Eppendorf concentrator plus) to remove methanol and acetone. The Reddish-pink colored pellets were finally dissolved in 200 μl of 100% methanol. UV spectra of extracted solutions were recorded at 200–700 nm range using a spectrophotometer (CECIL CE7500).

### Enzymatic Assay on a Starch Agar Plate

The α-amylase activity of the heterologously expressed Amy2 was measured using a starch agar plate assay. 1 gm of starch agar was dissolved in 100 ml of distilled water and poured into petri-plates. 50 μl of 5 μM purified Amy2 was loaded into starch agar wells and incubated overnight at 37°C. The activity was checked by staining the plates with an iodine solution [15% solution containing 5% (wt/v) I_2_ and 10% (wt/v) KI].

### Biochemical Characterization of Amy2

Biochemical characterization of the enzyme was performed by the iodine-starch method. The reaction solution of 50 μl contained 1.0 ISU/ml enzyme and 0.2% (w/v) starch in reaction buffer (20 mM Tris–HCl, 250 mM NaCl and 10 mM CaCl_2,_ pH 7.4). *A*_660_ was measured by standard enzyme assay. The effect of temperature on enzyme activity was determined by incubating the enzyme at the temperature range of 5–75°C at 10°C intervals for 30 mins. Optimum pH for enzyme activity was determined by measuring the activity at 37°C for 30 min under different pH conditions at an interval of 1.0 pH unit. Sodium acetate buffer was used for pH 3–5, phosphate buffer was used for pH 6–7, Tris buffer was used for pH 8–9, and sodium bicarbonate buffer was used for pH 10–11. Effect of salt concentration was observed by examining the activity at different NaCl concentration (0–4 M) at 37°C for 30 min. The data presented is an average of three independent experiments.

### Evolutionary Relationships of Taxa

The evolutionary history was inferred using the Neighbor-Joining method (Saitou and Nei, 1987). The optimal tree with the sum of branch length = 0.23099238 is shown. The percentage of replicate trees in which the associated taxa clustered together in the bootstrap test (1500 replicates) is shown next to the branches. The tree is drawn to scale, with branch lengths in the same units as those of the evolutionary distances used to infer the phylogenetic tree. The evolutionary distances were computed using the Maximum Composite Likelihood method and are in the units of the number of base substitutions per site. This analysis involved 16 nucleotide sequences. All ambiguous positions were removed for each sequence pair (pairwise deletion option). There were a total of 1483 positions in the final dataset. Evolutionary analyses were conducted in software suite MEGA X (Kumar et al., 2018).

## Data Availability Statement

Publicly available datasets were analyzed in this study. This data can be found here: GCA_900112175.1, GCA_900115785.1, GCA_000172995.2, GCA_000337835.1, GCA_000337815.1, GCA_000306765.2, GCA_000337795.1, GCA_000336815.1, GCA_000336955.1, GCA_001190965.1, GCA_000336755.1, GCA_000336735.1, GCA_000336795.1, GCA_000025685.1, GCA_001368915.1, and GCA_000337095.1.

## Author Contributions

KT, SP, and DV conceived the study. KT, SP, and AP coordinated the study. SP provided the strains. KT and DV designed experiments, analyzed the data, and wrote the manuscript with inputs from other co-authors. DV, GV, and CS performed experiments. All authors reviewed the results and approved the final version of the manuscript.

## Conflict of Interest

The authors declare that the research was conducted in the absence of any commercial or financial relationships that could be construed as a potential conflict of interest.

## Footnotes

1 https://crispr.i2bc.paris-saclay.fr/Server/

## References

[B1] Abe A. Yoshida H. Tonozuka T. Sakano Y. Kamitori S. (2005). Complexes of *Thermoactinomyces vulgaris* R-47 alpha-amylase 1 and pullulan model oligossacharides provide new insight into the mechanism for recognizing substrates with alpha-(1,6) glycosidic linkages. *FEBS J.* 272 6145–6153. 10.1111/j.1742-4658.2005.05013.x 16302977

[B2] Alikhan N. F. Petty N. K. Ben Zakour N. L. Beatson S. A. (2011). BLAST ring image generator (BRIG): simple prokaryote genome comparisons. *BMC Genomics* 12:402. 10.1186/1471-2164-12-402 21824423 PMC3163573

[B3] Amoozegar M. A. Safarpour A. Noghabi K. A. Bakhtiary T. Ventosa A. (2019). Halophiles and their vast potential in biofuel production. *Front. Microbiol.* 10:1895. 10.3389/fmicb.2019.01895 31507545 PMC6714587

[B4] Anderson I. Scheuner C. Goker M. Mavromatis K. Hooper S. D. Porat I. (2011). Novel insights into the diversity of catabolic metabolism from ten haloarchaeal genomes. *PLoS One* 6:e20237. 10.1371/journal.pone.0020237 21633497 PMC3102087

[B5] Antranikian G. (1992). *Microbial Degradation of Natural Products*, ed. Winkelmann G. Weinheim: VCH.

[B6] Aziz R. K. Bartels D. Best A. A. Dejongh M. Disz T. Edwards R. A. (2008). The RAST server: rapid annotations using subsystems technology. *BMC Genomics* 9:75. 10.1186/1471-2164-9-75 18261238 PMC2265698

[B7] Bajpai B. Chaudhary M. Saxena J. (2015). Production and Characterization of alpha-amylase from an extremely halophilic archaeon, haloferax sp. *HA*10. *Food Technol. Biotechnol.* 53 11–17. 10.17113/ftb.53.01.15.3824 27904327 PMC5068432

[B8] Becker E. A. Seitzer P. M. Tritt A. Larsen D. Krusor M. Yao A. I. (2014). Phylogenetically driven sequencing of extremely halophilic archaea reveals strategies for static and dynamic osmo-response. *PLoS Genet.* 10:e1004784. 10.1371/journal.pgen.1004784 25393412 PMC4230888

[B9] Bernstein F. C. Koetzle T. F. Williams G. J. Meyer E. F. Jr. Brice M. D. (1977). The protein data bank. A computer-based archival file for macromolecular structures. *Eur. J. Biochem.* 80 319–324. 10.1111/j.1432-1033.1977.tb11885.x 923582

[B10] Boel E. Brady L. Brzozowski A. M. Derewenda Z. Dodson G. G. Jensen V. J. (1990). Calcium binding in alpha-amylases: an X-ray diffraction study at 2.1*-*A resolution of two enzymes from *Aspergillus*. *Biochemistry* 29 6244–6249. 10.1021/bi00478a019 2207069

[B11] Borchert E. Knobloch S. Dwyer E. Flynn S. Jackson S. A. Johannsson R. (2017). Biotechnological potential of cold adapted *Pseudoalteromonas* spp. Isolated from ‘Deep Sea’ Sponges. *Mar. Drugs* 15:184. 10.3390/md15060184 28629190 PMC5484134

[B12] Bowers K. J. Mesbah N. M. Wiegel J. (2009). Biodiversity of poly-extremophilic Bacteria: does combining the extremes of high salt, alkaline pH and elevated temperature approach a physico-chemical boundary for life? *Saline Syst.* 5:9. 10.1186/1746-1448-5-9 19930649 PMC2785825

[B13] Bowers K. J. Wiegel J. (2011). Temperature and pH optima of extremely halophilic archaea: a mini-review. *Extremophiles* 15 119–128. 10.1007/s00792-010-0347-y 21340748

[B14] Britton G. (1995). Structure and properties of carotenoids in relation to function. *FASEB J.* 9 1551–1558. 10.1096/fasebj.9.15.8529834 8529834

[B15] Burns D. G. Janssen P. H. Itoh T. Kamekura M. Li Z. Jensen G. (2007). *Haloquadratum* walsbyi gen. nov., sp. nov., the square haloarchaeon of Walsby, isolated from saltern crystallizers in Australia and Spain. *Int. J. Syst. Evol. Microbiol.* 57 387–392. 10.1099/ijs.0.64690-0 17267984

[B16] Capes M. D. Dassarma P. Dassarma S. (2012). The core and unique proteins of haloarchaea. *BMC Genomics* 13:39. 10.1186/1471-2164-13-39 22272718 PMC3287961

[B17] Chaudhari N. M. Gupta V. K. Dutta C. (2016). BPGA- an ultra-fast pan-genome analysis pipeline. *Sci. Rep.* 6:24373. 10.1038/srep24373 PMC4829868 27071527

[B18] Chen S. Liu H. C. Zhou J. Xiang H. (2016). *Halorubrum pallidum* sp. nov., an extremely halophilic archaeon isolated from a subterranean rock salt. *Int. J. Syst. Evol. Microbiol.* 66 2980–2986. 10.1099/ijsem.0.001129 27150166

[B19] Chi M. C. Chen Y. H. Wu T. J. Lo H. F. Lin L. L. (2010). Engineering of a truncated alpha-amylase of *Bacillus* sp. strain TS-23 for the simultaneous improvement of thermal and oxidative stabilities. *J. Biosci. Bioeng.* 109 531–538. 10.1016/j.jbiosc.2009.11.012 20471589

[B20] Chun J. Lee J. H. Jung Y. Kim M. Kim S. Kim B. K. (2007). EzTaxon: a web-based tool for the identification of prokaryotes based on 16S ribosomal RNA gene sequences. *Int. J. Syst. Evol. Microbiol.* 57 2259–2261. 10.1099/ijs.0.64915-0 17911292

[B21] Cibrario A. Peanne C. Lailheugue M. Campbell-Sills H. Dols-Lafargue M. (2016). Carbohydrate metabolism in *Oenococcus oeni*: a genomic insight. *BMC Genomics* 17:984. 10.1186/s12864-016-3338-2 27905883 PMC5131533

[B22] Cowan S. T. Steel K. J. (1965). Manual for the identification of medical bacteria. *J. Clin. Pathol.* 46:975.

[B23] DeMaere M. Z. Williams T. J. Allen M. A. Brown M. V. Gibson J. A. Rich J. (2013). High level of intergenera gene exchange shapes the evolution of haloarchaea in an isolated Antarctic lake. *Proc. Natl. Acad. Sci. U.S.A.* 110 16939–16944. 10.1073/pnas.1307090110 24082106 PMC3801024

[B24] Dong G. Vieille C. Savchenko A. Zeikus J. G. (1997). Cloning, sequencing, and expression of the gene encoding extracellular alpha-amylase from *Pyrococcus furiosus* and biochemical characterization of the recombinant enzyme. *Appl. Environ. Microbiol.* 63 3569–3576. 10.1128/aem.63.9.3569-3576.1997 9293008 PMC168662

[B25] Falb M. Muller K. Konigsmaier L. Oberwinkler T. Horn P. Von Gronau S. (2008). Metabolism of halophilic archaea. *Extremophiles* 12 177–196. 18278431 10.1007/s00792-008-0138-x PMC2262144

[B26] Felsenstein J. (1985). Confidence limits on phylogenies: an approach using the bootstrap. *Evolution* 39 783–791. 10.1111/j.1558-5646.1985.tb00420.x 28561359

[B27] Fiedor J. Burda K. (2014). Potential role of carotenoids as antioxidants in human health and disease. *Nutrients* 6 466–488. 10.3390/nu6020466 24473231 PMC3942711

[B28] Flowers T. J. Colmer T. D. (2015). Plant salt tolerance: adaptations in halophytes. *Ann. Bot.* 115 327–331. 10.1093/aob/mcu267 25844430 PMC4332615

[B29] Giani M. Garbayo I. Vilchez C. Martinez-Espinosa R. M. (2019). Haloarchaeal carotenoids: healthy novel compounds from extreme environments. *Mar. Drugs* 17:524. 10.3390/md17090524 31500208 PMC6780574

[B30] Gilbert L. A. Larson M. H. Morsut L. Liu Z. Brar G. A. Torres S. E. (2013). CRISPR-mediated modular RNA-guided regulation of transcription in eukaryotes. *Cell* 154 442–451. 10.1016/j.cell.2013.06.044 23849981 PMC3770145

[B31] Grant W. (2001). Class III. Halobacteria class nov. *Bergey’s Manual Syst. Bacteriol.* 1 294–301.

[B32] Grant W. Larsen H. (1989). Extremely halophilic archaeobacteria, order Halobacteriales ord. *nov*. *Bergey’s Manual Syst. Bacteriol.* 3 2216–2228.

[B33] Grissa I. Vergnaud G. Pourcel C. (2007). CRISPRFinder: a web tool to identify clustered regularly interspaced short palindromic repeats. *Nucleic Acids Res.* 35 W52–W57. 17537822 10.1093/nar/gkm360 PMC1933234

[B34] Gruber C. Legat A. Pfaffenhuemer M. Radax C. Weidler G. Busse H. J. (2004). *Halobacterium noricense* sp. nov., an archaeal isolate from a bore core of an alpine Permian salt deposit, classification of *Halobacterium* sp. NRC-1 as a strain of *H. salinarum* and emended description of *H. salinarum*. *Extremophiles* 8 431–439. 10.1007/s00792-004-0403-6 15290323

[B35] Gunde-Cimerman N. Oren A. Plemenitas A. (2005). *Adaptation to Life at High Salt Concentrations in Archaea, Bacteria and Eukarya.* New York, NY: Kluwer Academic Publishers, 9.

[B36] Gupta R. Gigras P. Mohapatra H. Goswami V. K. Chauhan B. (2003). Microbial α-amylases: a biotechnological perspective. *Process Biochem.* 38 1599–1616. 10.1016/s0032-9592(03)00053-0

[B37] Horvath P. Barrangou R. (2010). CRISPR/Cas, the immune system of bacteria and archaea. *Science* 327 167–170. 10.1126/science.1179555 20056882

[B38] Hsiu J. Fischer E. H. Stein E. A. (1964). Alpha-amylases as calcium-metalloenzymes. Ii. Calcium and the catalytic activity. *Biochemistry* 3 61–66. 10.1021/bi00889a011 14114506

[B39] Hutcheon G. W. Vasisht N. Bolhuis A. (2005). Characterisation of a highly stable alpha-amylase from the halophilic archaeon Haloarcula hispanica. *Extremophiles* 9 487–495. 10.1007/s00792-005-0471-2 16075161

[B40] Javor B. J. (2012). *Hypersaline Environments: Microbiology and Biogeochemistry.* Berlin: Springer Science & Business Media.

[B41] Karray F. Ben Abdallah M. Kallel N. Hamza M. Fakhfakh M. Sayadi S. (2018). Extracellular hydrolytic enzymes produced by halophilic bacteria and archaea isolated from hypersaline lake. *Mol. Biol. Rep.* 45 1297–1309. 10.1007/s11033-018-4286-5 30062501

[B42] Kelley L. A. Mezulis S. Yates C. M. Wass M. N. Sternberg M. J. (2015). The Phyre2 web portal for protein modeling, prediction and analysis. *Nat. Protoc.* 10 845–858. 10.1038/nprot.2015.053 25950237 PMC5298202

[B43] Kim Y. B. Kim J. Y. Song H. S. Lee C. Ahn S. W. Lee S. H. (2018). Novel haloarchaeon Natrinema thermophila having the highest growth temperature among haloarchaea with a large genome size. *Sci. Rep.* 8:7777. 10.1038/s41598-018-25887-7 PMC5958107 29773867

[B44] Kumar S. Stecher G. Li M. Knyaz C. Tamura K. (2018). MEGA X: molecular evolutionary genetics analysis across computing platforms. *Mol. Biol. Evol.* 35 1547–1549. 10.1093/molbev/msy096 29722887 PMC5967553

[B45] Lányi B. (1988). 1 Classical and rapid identification methods for medically important bacteria. *Methods Microbiol.* 19 1–67. 10.1016/s0580-9517(08)70407-0

[B46] Littlechild J. A. (2015). Archaeal enzymes and applications in industrial biocatalysts. *Archaea* 2015:147671. 10.1155/2015/147671 PMC4606452 26494981

[B47] Lombard V. Golaconda Ramulu H. Drula E. Coutinho P. M. Henrissat B. (2014). The carbohydrate-active enzymes database (CAZy) in 2013. *Nucleic Acids Res.* 42 D490–D495. 24270786 10.1093/nar/gkt1178 PMC3965031

[B48] Machius M. Wiegand G. Huber R. (1995). Crystal structure of calcium-depleted *Bacillus licheniformis* alpha-amylase at 2.2 A resolution. *J. Mol. Biol.* 246 545–559. 7877175 10.1006/jmbi.1994.0106

[B49] Maier L.-K. Alkhnbashi O. S. Backofen R. Marchfelder A. (2017). “CRISPR and Salty: CRISPR-Cas Systems in Haloarchaea,” in *RNA Metabolism and Gene Expression in Archaea*, *Nucleic Acids and Molecular Biology*, Vol. 32, ed. Clouet-d’Orval B. (Cham: Springer), 243–269. 10.1007/978-3-319-65795-0_11

[B50] Maier L. K. Stachler A. E. Brendel J. Stoll B. Fischer S. Haas K. A. (2019). The nuts and bolts of the Haloferax CRISPR-Cas system I-B. *RNA Biol.* 16 469–480. 10.1080/15476286.2018.1460994 29649958 PMC6546412

[B51] Makarova K. S. Koonin E. V. (2015). Annotation and classification of CRISPR-Cas systems. *Methods Mol. Biol.* 1311 47–75. 10.1007/978-1-4939-2687-9_4 25981466 PMC5901762

[B52] Mata-Gomez L. C. Montanez J. C. Mendez-Zavala A. Aguilar C. N. (2014). Biotechnological production of carotenoids by yeasts: an overview. *Microb. Cell Fact.* 13:12. 10.1186/1475-2859-13-12 24443802 PMC3922794

[B53] Medema M. H. Blin K. Cimermancic P. De Jager V. Zakrzewski P. Fischbach M. A. (2011). antiSMASH: rapid identification, annotation and analysis of secondary metabolite biosynthesis gene clusters in bacterial and fungal genome sequences. *Nucleic Acids Res.* 39 W339–W346. 21672958 10.1093/nar/gkr466 PMC3125804

[B54] Minegishi H. Kamekura M. Itoh T. Echigo A. Usami R. Hashimoto T. (2010). Further refinement of the phylogeny of the Halobacteriaceae based on the full-length RNA polymerase subunit B’(rpoB’) gene. *Int. J. Syst. Evol. Microbiol.* 60 2398–2408. 10.1099/ijs.0.017160-0 19946058

[B55] Montalvo-Rodriguez R. Vreeland R. H. Oren A. Kessel M. Betancourt C. Lopez-Garriga J. (1998). *Halogeometricum borinquense* gen. nov., sp. nov., a novel halophilic archaeon from Puerto Rico. *Int. J. Syst. Bacteriol.* 48(Pt 4), 1305–1312. 10.1099/00207713-48-4-1305 9828431

[B56] Montero-Lobato Z. Ramos-Merchante A. Fuentes J. L. Sayago A. Fernandez-Recamales A. Martinez-Espinosa R. M. (2018). Optimization of growth and carotenoid production by haloferax mediterranei using response surface methodology. *Mar. Drugs* 16:372. 10.3390/md16100372 30304770 PMC6213265

[B57] Mormile M. R. Biesen M. A. Gutierrez M. C. Ventosa A. Pavlovich J. B. Onstott T. C. (2003). Isolation of *Halobacterium salinarum* retrieved directly from halite brine inclusions. *Environ. Microbiol.* 5 1094–1102. 10.1046/j.1462-2920.2003.00509.x 14641589

[B58] Nagata S. Hyde C. C. Miles E. W. (1989). The alpha subunit of tryptophan synthase. Evidence that aspartic acid 60 is a catalytic residue and that the double alteration of residues 175 and 211 in a second-site revertant restores the proper geometry of the substrate binding site. *J. Biol. Chem.* 264 6288–6296. 2649498

[B59] Naghoni A. Emtiazi G. Amoozegar M. A. Cretoiu M. S. Stal L. J. Etemadifar Z. (2017). Microbial diversity in the hypersaline Lake Meyghan, Iran. *Sci. Rep.* 7:11522. 10.1038/s41598-017-11585-3 PMC5599592 28912589

[B60] Narsing Rao M. P. Xiao M. Li W. J. (2017). Fungal and bacterial pigments: secondary metabolites with wide applications. *Front. Microbiol.* 8:1113. 10.3389/fmicb.2017.01113 28690593 PMC5479939

[B61] Naziri D. Hamidi M. Hassanzadeh S. Tarhriz V. Maleki Zanjani B. Nazemyieh H. (2014). Analysis of carotenoid production by *Halorubrum* sp. TBZ126; an extremely Halophilic Archeon from Urmia Lake. *Adv. Pharm. Bull.* 4 61–67. 24409411 10.5681/apb.2014.010 PMC3885371

[B62] Neilands J. B. (1995). Siderophores: structure and function of microbial iron transport compounds. *J. Biol. Chem.* 270 26723–26726. 10.1074/jbc.270.45.26723 7592901

[B63] Ollivier B. Caumette P. Garcia J. L. Mah R. A. (1994). Anaerobic bacteria from hypersaline environments. *Microbiol. Rev.* 58 27–38. 10.1128/mmbr.58.1.27-38.1994 8177169 PMC372951

[B64] Onodera M. Yatsunami R. Tsukimura W. Fukui T. Nakasone K. Takashina T. (2013). Gene analysis, expression, and characterization of an intracellular alpha-amylase from the extremely halophilic archaeon *Haloarcula japonica*. *Biosci. Biotechnol. Biochem.* 77 281–288. 10.1271/bbb.120693 23391916

[B65] Oren A. (2002). Molecular ecology of extremely halophilic Archaea and Bacteria. *FEMS Microbiol. Ecol.* 39 1–7. 10.1111/j.1574-6941.2002.tb00900.x 19709178

[B66] Oren A. (2010). Industrial and environmental applications of halophilic microorganisms. *Environ. Technol.* 31 825–834. 10.1080/09593330903370026 20662374

[B67] Oren A. (2012). The function of gas vesicles in halophilic archaea and bacteria: theories and experimental evidence. *Life* 3 1–20. 10.3390/life3010001 25371329 PMC4187190

[B68] Oren A. Ventosa A. Grant W. (1997). Proposed minimal standards for description of new taxa in the order Halobacteriales. *Int. J. Syst. Evol. Microbiol.* 47 233–238. 10.1099/00207713-47-1-233

[B69] Papke R. T. Corral P. Ram-Mohan N. De La Haba R. R. Sanchez-Porro C. Makkay A. (2015). Horizontal gene transfer, dispersal and haloarchaeal speciation. *Life* 5 1405–1426. 10.3390/life5021405 25997110 PMC4500145

[B70] Perez-Pomares F. Bautista V. Ferrer J. Pire C. Marhuenda-Egea F. C. Bonete M. J. (2003). Alpha-amylase activity from the halophilic archaeon *Haloferax mediterranei*. *Extremophiles* 7 299–306. 10.1007/s00792-003-0327-6 12910390

[B71] Pettit R. K. (2011). Culturability and secondary metabolite diversity of extreme microbes: expanding contribution of deep sea and deep-sea vent microbes to natural product discovery. *Mar. Biotechnol.* 13 1–11. 10.1007/s10126-010-9294-y 20437069

[B72] Pietra F. (1997). Secondary metabolites from marine microorganisms: bacteria, protozoa, algae and fungi. Achievements and prospects. *Nat. Prod. Rep.* 14 453–464. 9364777 10.1039/np9971400453

[B73] Prakash O. Jaiswal N. (2010). alpha-Amylase: an ideal representative of thermostable enzymes. *Appl. Biochem. Biotechnol.* 160 2401–2414. 10.1007/s12010-009-8735-4 19763902

[B74] Radax C. Gruber C. Stan-Lotter H. (2001). Novel haloarchaeal 16S rRNA gene sequences from Alpine Permo-Triassic rock salt. *Extremophiles* 5 221–228. 10.1007/s007920100192 11523891

[B75] Rodrigo-Banos M. Garbayo I. Vilchez C. Bonete M. J. Martinez-Espinosa R. M. (2015). Carotenoids from Haloarchaea and their potential in biotechnology. *Mar. Drugs* 13 5508–5532. 10.3390/md13095508 26308012 PMC4584337

[B76] Saitou N. Nei M. (1987). The neighbor-joining method: a new method for reconstructing phylogenetic trees. *Mol. Biol. Evol.* 4 406–425. 3447015 10.1093/oxfordjournals.molbev.a040454

[B77] Santorelli M. Maurelli L. Pocsfalvi G. Fiume I. Squillaci G. La Cara F. (2016). Isolation and characterisation of a novel alpha-amylase from the extreme haloarchaeon *Haloterrigena turkmenica*. *Int. J. Biol. Macromol.* 92 174–184. 10.1016/j.ijbiomac.2016.07.001 27377461

[B78] Sarian F. D. Janecek S. Pijning T. Ihsanawati Nurachman Z. Radjasa O. K. (2017). A new group of glycoside hydrolase family 13 alpha-amylases with an aberrant catalytic triad. *Sci. Rep.* 7:44230. 10.1038/srep44230 PMC5347038 28287181

[B79] Sasser M. (1990). *Identification of Bacteria by Gas Chromatography of Cellular Fatty Acids.* Newark, DE: MIDI Inc.

[B80] Schiraldi C. Giuliano M. De Rosa M. (2002). Perspectives on biotechnological applications of archaea. *Archaea* 1 75–86. 10.1155/2002/436561 15803645 PMC2685559

[B81] Schubert B. A. Lowenstein T. K. Timofeeff M. N. Parker M. A. (2010). Halophilic Archaea cultured from ancient halite, Death Valley, California. *Environ. Microbiol.* 12 440–454. 10.1111/j.1462-2920.2009.02086.x 19840101

[B82] Shigechi H. Koh J. Fujita Y. Matsumoto T. Bito Y. Ueda M. (2004). Direct production of ethanol from raw corn starch via fermentation by use of a novel surface-engineered yeast strain codisplaying glucoamylase and alpha-amylase. *Appl. Environ. Microbiol.* 70 5037–5040. 10.1128/aem.70.8.5037-5040.2004 15294847 PMC492352

[B83] Singh A. Singh A. K. (2017). Haloarchaea: worth exploring for their biotechnological potential. *Biotechnol. Lett.* 39 1793–1800. 10.1007/s10529-017-2434-y 28900776

[B84] Sivakumar N. Li N. Tang J. W. Patel B. K. Swaminathan K. (2006). Crystal structure of AmyA lacks acidic surface and provide insights into protein stability at poly-extreme condition. *FEBS Lett.* 580 2646–2652. 10.1016/j.febslet.2006.04.017 16647060

[B85] Sivaramakrishnan S. Gangadharan D. Nampoothiri K. M. Soccol C. R. Pandey A. (2006). a-Amylases from microbial sources–an overview on recent developments. *Food Technol. Biotechnol.* 44 173–184.

[B86] Smibert R. Krieg N. Gerhardt P. Murray R. Wood W. (1994). *Methods for General and Molecular Bacteriology.* Washington DC: American Society for Microbiology.

[B87] Soppa J. (2006). From genomes to function: haloarchaea as model organisms. *Microbiology* 152 585–590. 10.1099/mic.0.28504-0 16514139

[B88] Stan-Lotter H. Fendrihan S. (2015). Halophilic Archaea: life with desiccation, radiation and oligotrophy over geological times. *Life* 5 1487–1496. 10.3390/life5031487 26226005 PMC4598649

[B89] Tamura K. Nei M. Kumar S. (2004). Prospects for inferring very large phylogenies by using the neighbor-joining method. *Proc. Natl. Acad. Sci. U.S.A.* 101 11030–11035. 10.1073/pnas.0404206101 15258291 PMC491989

[B90] Tettelin H. Masignani V. Cieslewicz M. J. Donati C. Medini D. Ward N. L. (2005). Genome analysis of multiple pathogenic isolates of Streptococcus agalactiae: implications for the microbial “pan-genome”. *Proc. Natl. Acad. Sci. U.S.A.* 102 13950–13955. 16172379 10.1073/pnas.0506758102 PMC1216834

[B91] Torreblanca M. Rodriguez-Valera F. Juez G. Ventosa A. Kamekura M. Kates M. (1986). Classification of non-alkaliphilic halobacteria based on numerical taxonomy and polar lipid composition, and description of *Haloarcula* gen. nov. and *Haloferax* gen. nov. *Syst. Appl. Microbiol.* 8 89–99. 10.1016/s0723-2020(86)80155-2

[B92] Verma D. K. Baral I. Kumar A. Prasad S. E. Thakur K. G. (2019). Discovery of bacteriorhodopsins in Haloarchaeal species isolated from Indian solar salterns: deciphering the role of the N-terminal residues in protein folding and functional expression. *Microb. Biotechnol.* 12 434–446. 10.1111/1751-7915.13359 30648822 PMC6465532

[B93] Vernikos G. Medini D. Riley D. R. Tettelin H. (2015). Ten years of pan-genome analyses. *Curr. Opin. Microbiol.* 23 148–154. 10.1016/j.mib.2014.11.016 25483351

[B94] Vershinin A. (1999). Biological functions of carotenoids–diversity and evolution. *Biofactors* 10 99–104. 10.1002/biof.5520100203 10609869

[B95] Vilchez C. Forjan E. Cuaresma M. Bedmar F. Garbayo I. Vega J. M. (2011). Marine carotenoids: biological functions and commercial applications. *Mar. Drugs* 9 319–333. 10.3390/md9030319 21556162 PMC3083653

[B96] Vos M. Didelot X. (2009). A comparison of homologous recombination rates in bacteria and archaea. *ISME J.* 3 199–208. 10.1038/ismej.2008.93 18830278

[B97] Wang S. Zheng Z. Zou H. Li N. Wu M. (2019). Characterization of the secondary metabolite biosynthetic gene clusters in archaea. *Comput. Biol. Chem.* 78 165–169. 10.1016/j.compbiolchem.2018.11.019 30530297

[B98] Werries E. Muller F. (1986). Studies on the substrate specificity of alpha- and beta-amylase of Entamoeba histolytica. *Mol. Biochem. Parasitol.* 18 211–221. 10.1016/0166-6851(86)90039-3 2421162

[B99] Winters Y. D. Lowenstein T. K. Timofeeff M. N. (2015). Starvation-survival in Haloarchaea. *Life* 5 1587–1609. 10.3390/life5041587 26569313 PMC4695838

[B100] Wolf Y. I. Makarova K. S. Yutin N. Koonin E. V. (2012). Updated clusters of orthologous genes for Archaea: a complex ancestor of the Archaea and the byways of horizontal gene transfer. *Biol. Direct.* 7:46. 10.1186/1745-6150-7-46 23241446 PMC3534625

[B101] Wu S. Zhu Z. Fu L. Niu B. Li W. (2011). WebMGA: a customizable web server for fast metagenomic sequence analysis. *BMC Genomics* 12:444. 10.1186/1471-2164-12-444 21899761 PMC3180703

[B102] Yang Y. Yatsunami R. Ando A. Miyoko N. Fukui T. Takaichi S. (2015). Complete biosynthetic pathway of the C50 carotenoid bacterioruberin from lycopene in the extremely halophilic archaeon *Haloarcula japonica*. *J. Bacteriol.* 197 1614–1623. 10.1128/jb.02523-14 25712483 PMC4403650

[B103] Yatsunami R. Ando A. Yang Y. Takaichi S. Kohno M. Matsumura Y. (2014). Identification of carotenoids from the extremely halophilic archaeon *Haloarcula japonica*. *Front. Microbiol.* 5:100. 10.3389/fmicb.2014.00100 24672517 PMC3956123

[B104] Yoon S. H. Ha S. M. Kwon S. Lim J. Kim Y. Seo H. (2017). Introducing EzBioCloud: a taxonomically united database of 16S rRNA gene sequences and whole-genome assemblies. *Int. J. Syst. Evol. Microbiol.* 67 1613–1617. 10.1099/ijsem.0.001755 28005526 PMC5563544

[B105] Zhang J. Sun Z. Sun P. Chen T. Chen F. (2014). Microalgal carotenoids: beneficial effects and potential in human health. *Food Funct.* 5 413–425. 24480814 10.1039/c3fo60607d

