# Supplementary material for: Biochemical and Taxonomic Characterization of Novel Haloarchaeal Strains and Purification of the Recombinant Halotolerant α-Amylase Discovered in the Isolate
Source: Front Microbiol. 2020 Sep 1;11:2082. doi: 10.3389/fmicb.2020.02082 (PMC7490331; doi:10.3389/fmicb.2020.02082)
Supplement: Supplementary file 1 [file Data_Sheet_1.docx]

**Supplementary Information**

**Biochemical and taxonomic characterization of novel Indian Haloarchaeal strains and purification of the recombinant halotolerant α-amylase discovered in the isolate**

Dipesh Kumar Verma^1^, Gunjan Vasudeva^2^, Chandni Sidhu^2^, Anil K. Pinnaka^2^, Senthil E. Prasad^3#^ and Krishan Gopal Thakur^1#^

^1^G. N. Ramachandran Protein Centre, Structural Biology Laboratory, Council of Scientific and Industrial Research-Institute of Microbial Technology (CSIR-IMTECH), Chandigarh-160036, India

^2^MTCC-Microbial Type Culture Collection & Gene Bank, CSIR-Institute of Microbial Technology, Chandigarh-160036, India.

^3^Biochemical Engineering Research and Process Development Centre, Council of Scientific and Industrial Research-Institute of Microbial Technology (CSIR-IMTECH), Chandigarh-160036, India

**Correspondence**

^#^Krishan Gopal Thakur

[Email: krishang@imtech.res.in](mailto:Email:%20krishang@imtech.res.in)

^#^Senthil E. Prasad

[Email: esprasad@imtech.res.in](mailto:Email:%20esprasad@imtech.res.in)

**

**

**Figure S1.**TEM imaging showing morphological variations observed in the haloarchaeal isolates wsp1, wsp3 and wsp4.

|  | **Metric** | **Value** |
| --- | --- | --- |
| **Wsp1** | *Haloferax* wsp1 genome length (bp) | 3,706,680 |
|  | *Haloferax volcanii* DS2 genome length (bp) | 4,010,640 |
|  | Average alignment length (bp) | 2,338,133 |
|  | OrthoANI value (%) | 97.90 |
|  | | |
| **Wsp3** | *Halogeometricum* wsp3 genome length (bp) | 2,818,260 |
|  | *H. borinquense* genome DSM 11551length (bp) | 3,943,320 |
|  | Average alignment length (bp) | 2,098,583 |
|  | OrthoANI value (%) | 97.34 |
|  | | |
| **Wsp4** | *Halogeometricum* wsp4 genome length (bp) | 3,991,260 |
|  | *H. borinquense* genome DSM 11551length (bp) | 3,943,320 |
|  | Average alignment length (bp) | 2,609,196 |
|  | OrthoANI value (%) | 96.94 |
|  | OrthoANI value (%) | 96.94 |

**Supplementary Table 1. Average nucleotide index**

| **Query genome** | **Reference genome** | **Distance** | **Prob. DDH** |
| --- | --- | --- | --- |
| *Haloferax* wsp1 | *H. volcanii* | 0.1668 | 95.82 |
| *Halogeometricum* wsp3 | *H. borinquense* | 0.1869 | 91.91 |
| *Halogeometricum* wsp4 | *H. borinquense* | 0.128 | 98.89 |

**Supplementary Table 2. DNA-DNA hybridization**

**Supplementary Table 3. CRISPR sequences found in the genome**

| **Strain Name** | **DR sequences** | **Spacer** |
| --- | --- | --- |
| **Wsp1** | GCTTCAACCCAACAAGGGTTCGTCTGAAAC | 41 |
|  | GCTTCAATCCCACAAGGGTTCGTCTGAAAC | 7 |
|  | GCTTCAACCCAACAAGGGTTCGTCTGAAAC | 19 |
|  | GCTTCAACCCCACAAGGGTTCGTCTGAAAC | 10 |
|  | GCTTCAACCCCACAAGGGTTCGTCTGAAAC | 19 |
|  | GCTTCAACCCCACAAGGGTTCGTCTGAAA | 34 |
| **Wsp3** | GCTTCAACCCCACAAGGGTTCGTCTGTAAC | 4 |
| **Wsp4** | GTTTCAGACGAACCCTTGTGGGATTGAAGC | 30 |
|  | GTTTCAGACGAACCCTTGTGGGATTGAAGC | 15 |
|  | GTTACAGACGAACCCTTGTGGGGTTGAAGC | 6 |
|  | CACCCCCCTCATTTCGTCTGTTAG | 4 |

**Supplementary Table 4.Genes involved in secondary metabolite biosynthesis**

| **Organism** | **Siderophore** | **Terpene** |
| --- | --- | --- |
| Wsp1 | 1 | 2 |
| Wsp3 | - | 2 |
| Wsp4 | 1 | 2 |
